# Evaluation of the Predictive Performance of Population Pharmacokinetic Models of Adalimumab in Patients with Inflammatory Bowel Disease

**DOI:** 10.3390/pharmaceutics13081244

**Published:** 2021-08-12

**Authors:** Silvia Marquez-Megias, Amelia Ramon-Lopez, Patricio Más-Serrano, Marcos Diaz-Gonzalez, Maria Remedios Candela-Boix, Ricardo Nalda-Molina

**Affiliations:** 1 School of Pharmacy, Miguel Hernández University, 03550 San Juan de Alicante, Spain; silvia.marquez@goumh.umh.es (S.M.-M.); mas_pat@gva.es (P.M.-S.); jnalda@umh.es (R.N.-M.); 2 Alicante Institute for Health and Biomedical Research (ISABIAL-FISABIO Foundation), 03010 Alicante, Spain; diaz_marcosgon@gva.es; 3 Clinical Pharmacokinetics Unit, Pharmacy Department, Alicante University General Hospital, 03010 Alicante, Spain; 4 Virgen de la Salud General Hospital of Elda, 03600 Elda, Spain; candela_marboi@gva.es

**Keywords:** pharmacokinetics, drug monitoring, adalimumab, inflammatory bowel diseases, Crohn’s disease, colitis, ulcerative

## Abstract

Adalimumab is a monoclonal antibody used for inflammatory bowel disease. Due to its considerably variable pharmacokinetics, the loss of response and the development of anti-antibodies, it is highly recommended to use a model-informed precision dosing approach. The aim of this study is to evaluate the predictive performance of different population-pharmacokinetic models of adalimumab for inflammatory bowel disease to determine the pharmacokinetic model(s) that best suit our population to use in the clinical routine. A retrospective observational study with 134 patients was conducted at the General University Hospital of Alicante between 2014 and 2019. Model adequacy of each model was evaluated by the distribution of the individual pharmacokinetic parameters and the NPDE plots whereas predictive performance was assessed by calculating bias and precision. Moreover, stochastic simulations were performed to optimize the maintenance doses in the clinical protocols, to reach the target of 8 mg/L in at least 75% of the population. Two population-pharmacokinetic models were selected out of the six found in the literature which performed better in terms of adequacy and predictive performance. The stochastic simulations suggested the benefits of increasing the maintenance dose in protocol to reach the 8 mg/L target.

## 1. Introduction

Crohn’s disease (CD) and ulcerative colitis (UC) are chronic inflammatory bowel diseases (IBD) characterized by the intermittent destructive inflammation of the intestinal tract associated with significant morbidity, high burden of hospitalization and a severe impact on the quality of life of patients. There are several pharmacological alternatives available, including corticosteroids, immunosuppressive agents (methotrexate or azathioprine) and monoclonal antibodies that have shown clinical response in the treatment of these diseases [1,2,3].

Adalimumab is a human monoclonal antibody that binds specifically to the tumor necrosis factor (TNF) and neutralizes its biological function, decreasing the process of inflammation. Adalimumab is effective for induction and maintenance of remission in patients with moderate-to-severe IBD older than 6 years who fail with corticosteroids, immunosuppressive agents or other biologic therapy [4,5,6].

Several published studies of adalimumab have shed light on the clinical relevance of individualized dosing. Historically, the empiric approach to adapt the adalimumab dosage consists of intensifying the treatment in patients with loss of response and later, if this fails, switching to another biological treatment. In the last decade, several studies have shown that some patients can experience a loss of response to adalimumab or can develop antibodies against adalimumab (AAA) after long periods of subtherapeutic drug levels [7,8,9,10,11,12,13,14]. However, most of the time, the serum concentration guide dosing was done through algorithms [15,16].

In this line, Model-Informed Precision Dosing (MIPD) is the approach based on the use of population PK (PopPK) models and prospective Bayesian approach to increase the homogeneity in the drug exposure in patients in order to improve outcomes of treatments by achieving the optimal balance between efficacy and toxicity for each individual patient [17]. IBD patients could benefit from dose optimization because adalimumab has highly variable pharmacokinetics (PK) [16,18].

Recently, a multicenter retrospective study showed that the potential importance of early monitoring levels of adalimumab and MIPD approach can prevent immunogenicity and achieve better long-term outcomes in terms of IBD-related surgery or hospitalization, lower risk of developing AAA or serious infusion reactions and also it proved to be more cost-effective in comparison to empirical and/or reactive dose optimization program dose escalation [19]. However, the selection of the appropriate PopPK model is fundamental to apply MIPD, especially when there are multiple models in the literature in patients with IBD. The structural model is defined, in most of them, as one-compartment model with linear kinetics in the absorption and elimination processes, although the value of the PopPK parameters, and the covariates included in the model, vary significantly. Therefore, the aim of this study is to evaluate the predictive performance of PopPK models of adalimumab found in literature, in patients with IBD to determine the pharmacokinetic model(s) best suited for our population to subsequently use it in the clinical setting using MIPD.

## 2. Materials and Methods

### 2.1. Literature Search

A systematic literature search was conducted of databases in the field of Health Sciences: MEDLINE (via PubMed), Embase and Scopus. To define the search terms, the Medical Subject Headings (MeSH), a thesaurus developed by the U.S. National Library of Medicine, was used. The MeSH descriptors “Chron Disease”, “Colitis, Ulcerative”, “adalimumab” and “pharmacokinetics” were considered suitable. Likewise, these terms, “inflammatory bowel diseases” and “pharmacokinetics” were used to query the databases using the title and abstract field (Title/Abstract). The search was performed from the first available date until May 2021 according to the characteristics of each database. Additionally, a manual search for population models was conducted by inspecting the bibliographies of relevant journal articles to minimize the number of unrecovered papers by the review.

The following search was used in Pubmed, and it was adapted to the other databases: (((((“Inflammatory Bowel Diseases”[Mesh]) OR (Inflammatory Bowel Diseases[Title/Abstract])) OR (((Crohn Disease[Title/Abstract]) OR (Crohn’s Disease[Title/Abstract])) OR (“Crohn Disease”[Mesh]))) OR ((ulcerative colitis[Title/Abstract]) OR (“Colitis, Ulcerative”[Mesh]))) AND ((Adalimumab[Title/Abstract]) OR (“Adalimumab”[Mesh]))) AND ((Pharmacokinetics[Title/Abstract]) OR (“Pharmacokinetics”[Mesh])).

The inclusion criteria were the following: original articles published in peer-reviewed journals, articles that describe a novel population pharmacokinetic model and pertinent works with the available complete text, which must be written in English or Spanish. Additionally, the full text of the document should be accessible and only one version of each document was included. The following were the exclusion criteria: articles that included different diseases to CD or UC and studies developed in animal models.

The following information was extracted from the articles: patient characteristics, model structure, typical PopPK parameters, inter-individual variability (IIV), residual variability (RV) and covariates.

### 2.2. Study Design

A retrospective observational study was conducted at the General University Hospital of Alicante, performed on patients diagnosed with IBD undergoing treatment with adalimumab and who followed a dose optimization program developed between 2014 and 2019.

### 2.3. Patients and Data Collection

Trough serum concentrations (TSC) were collected from patients diagnosed with moderate or severe IBD treated with adalimumab in General University Hospital of Alicante, Spain. The following inclusion criteria were applied: participants had to be diagnosed with IBD, treated with adalimumab, and there had to be at least two adalimumab TSC in their medical history. Exclusion criteria included patients treated with other monoclonal antibodies different to adalimumab like infliximab, vedolizumab and ustekinumab and subjects who were diagnosed with other autoimmune diseases different to IBD such as rheumatoid arthritis, psoriasis and ankylosing spondylitis.

Relevant data were collected from the medical records and included age, sex, height, body weight, lean body weight (LBW), body mass index (BMI), AAA status and AAA serum concentration, dose of adalimumab, adalimumab serum concentration, serum albumin levels, serum C-reactive protein (CRP) levels, fecal calprotectin (FCP), type of disease, use of concomitant immunomodulators and time of the event recorded. Missing values of continuous covariates were imputed by their expected mean values. Data were excluded from the analysis if there was uncertainty about any relevant information such as the time of dosing or the time of drug concentration measurement and the loss to follow-up during their treatment.

Serum adalimumab concentrations and AAA were measured using an enzyme-linked immunosorbent assay (LISA TRACKER Duo Drug + ADAb from TheraDiag^®^) with a limit of quantification established to be 0.1 mg/L. Patients were considered as positive for AAA if titers were above 10 mg/L on at least one occasion.

### 2.4. Evaluation of Model Adequacy

The first step in the evaluation of the different PopPk models found in the literature was the evaluation of the model adequacy by analyzing and comparing how the different PopPK models describe the studied population using all the available TSC in the dataset (full dataset). Models that show the greater systematic bias in the Empirical Bayesian estimate (EBEs) of the PK parameters, or in the Normalised Prediction Distribution Errors (NPDE) [20,21] will be discarded. Only the models that described properly our population will be used to evaluate the predictive performance later.

Therefore, the distribution of the EBEs of the PK parameters for each of the PopPk models was calculated after performing a post-hoc analysis using the full dataset. Then, this distribution would be compared with the theoretical distribution of these PK parameters according to each of the PopPK models.

On the other hand, any trends observed in the NPDE plots (e.g., cone-shaped graph) might indicate model misspecifications and inferior model adequacy.

### 2.5. Evaluation of Predictive Performance

The evaluation of predictive performance was only performed in those models which best describe the studied population, according to the evaluation of the model adequacy.

To evaluate the predictive performance, the individual predictions of the last TSC were estimated for each patient, using the EBEs. These last TSC concentrations, named “last observed TSC”, were left out and not used to calculate the EBEs. To evaluate the predictive performance, the bias and the precision were calculated with the last observed TSC by comparing them with their individual predictions calculated by each of the PK models. The predictive performance of the patients was evaluated considering two different scenarios; Scenario 1: The EBES were calculated from the previous TSC obtained from each patient 2 Scenario 2: The EBES were calculated from the two previous TSC of each patient. 

The mean prediction error (MPE, Equation (1)) and root mean square prediction error (RMSPE, Equation (2)) were calculated for bias and precision, respectively.

(1) $$\mathrm{MPE}=\frac{\sum^{\text{​}}\left(\hat{Y}-Y\right)}{n}$$

(2) $$\mathrm{RMSPE}=\sqrt{\frac{\sum^{\text{​}}{\left(\hat{Y}-Y\right)}^{2}}{n}}$$

In both equations *Y-hat* represents the model-predicted adalimumab concentration, *Y* represents the observed adalimumab concentration, and *n* is the number of observations.

A bootstrap of the data was performed to compare the statistical significance of the differences between bias and precision among the selected models. 

### 2.6. Clinical Impact

Stochastic simulations were performed to optimize the initial maintenance doses in the clinical protocols, in order to acquire the target TSC in at least 75% of the population. The dosage regimens that were simulated were 40 and 80 mg administered subcutaneously every week or every other week. The target TSC that were considered were 8 mg/L for clinical remission [18,22].

### 2.7. Software

The PopPK models found in the literature were implemented in NONMEM^®^ version 7.4 software package [23]. The posterior statistical analysis and graphics were performed using R software v4.0.3 [24], implemented in R-studio v1.3.1093 [25].

### 2.8. Ethical Considerations

#### 2.8.1. Ethics Approval

All studies were conducted in accordance with principles for human experimentation as defined in the Declaration of Helsinki and were approved by the Human Investigational Review Board of each study center.

#### 2.8.2. Consent

The need for written consent was waived because of the retrospective nature of the study.

## 3. Results

### 3.1. Literature Search

A total of 211 publications 72, 52 and 87 from PubMed, Embase and Scopus, respectively, from 2003 to 2021, were found and collected in the search of databases using the keywords mentioned in the methods section. After removing duplicate articles and applying the inclusion and exclusion criteria, six PopPK models [26,27,28,29,30,31] were selected. The models were numbered from 1 to 6 and are referred to as M1 to M6. All selected PopPK models were one-compartment models. Four of them included only trough levels of adalimumab (M2, M3, M4 and M5) whereas the others (M1 and M6) derived from complete profiles of serum concentrations of adalimumab. Five of the six models were developed using NONMEM^®^ software, while one model (M2) was developed using Monolix^®^ software. Further information can be found in Table 1.

Typical values for adalimumab apparent clearance (CL/F) in the studies ranged from 11.7 to 17.5 mL/h, with the lowest value being reported in studies performed with pediatric population (M3). The typical apparent volume of distribution (V/F) ranged from 4.07 to 13.5 L. The absorption rate constant (ka) was estimated in three models and fixed in the others. All models estimated the IIV (coefficient of variation [CV], in percent) associated with adalimumab CL/F, with values ranging from 16.4% to 65%. Three models (M1, M2 and M5) estimated the IIV of V/F ranged from 35.1% to 48%. The summary of the characteristics of each study is listed in Table 2.

### 3.2. Patients 

The dataset included 134 IBD patients in treatment with adalimumab with at least two TC. Baseline demographics, disease characteristics and missing values for the different covariates of the patient population are listed in Table 3. 75% of the patients are below 57 years old and 75 kg. Approximately 85% of the patients were diagnosed as CD and 8% of them developed AAA.

82 patients were treated subcutaneously with 160/80 mg and 18 with 80/40 mg at weeks 0/2 as an induction phase. For the rest, the information regarding the induction phase was not available in their medical histories. Following this phase, as a maintenance phase, all patients were treated with 40 mg of adalimumab every other week. A total of 398 TSC in the maintenance phase were available for the analysis, where 25.4% of these concentrations were over 8 mg/L, 46.3% between 3 and 8 mg/L and 28.3% below 3 mg/L in the first measure. AAA were detected in 11 patients. 73 patients were on a concomitant immunomodulator (azathioprine, 6 mercaptopurine, methotrexate or prednisone). 

The dosage regimen was increased to 40 mg every 10 days or 40 mg every week, on 31 and 70 dose adjustments, respectively. Similarly, the dosage regimen was increased to 80 mg every other week or 80 mg every week, on 7 and 11 dose adjustments, respectively. On the other hand, on 7 dose adjustments, the dosage regimen was decreased to 40 mg every 3 weeks, at any time during their treatment. In 36 patients the dosage regimen was maintained at 40 mg every other week.

### 3.3. Evaluation of Model Adequacy

**Figure 1:**
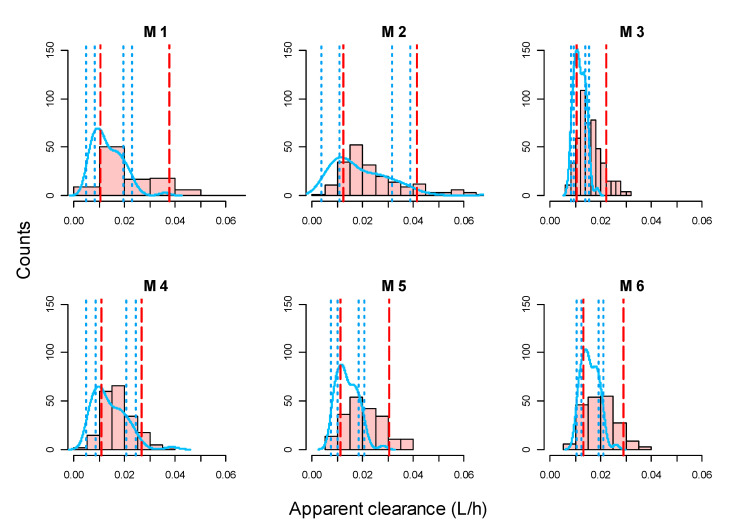
Histograms of EBEs for CL/F. Red dashed line; 20th and 80th percentile of EBEs CL/F; blue solid line represents the density of the simulated CL/F; blue dotted line, 95% confidence interval (CI) for the 20th and 80th percentiles of simulated CL/F. The M numbers represent the models described in Table 1.

**Figure 2:**
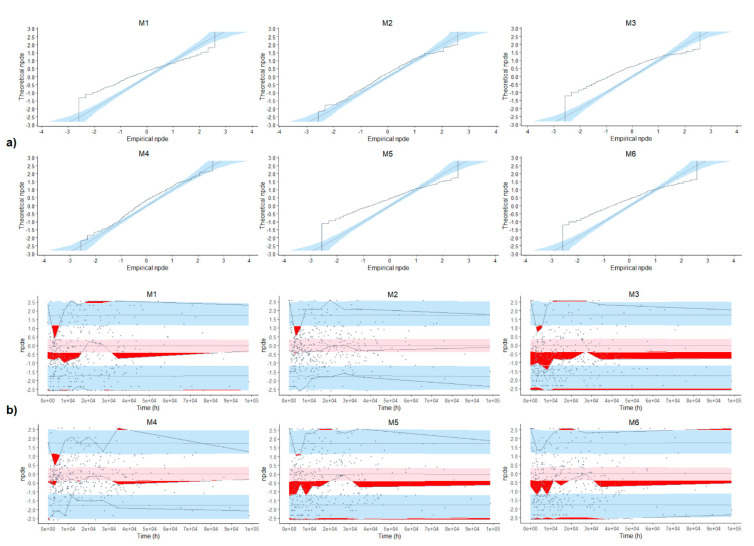
NPDE for each model. (**a**) Quantile-quantile plot of the npde versus the corresponding quantiles of a normal distribution. (**b**) Plot of npde versus time. Solid horizontal lines are the lines corresponding to 0, 5% and 95% critical values; gray solid lines, prediction intervals; blue-shaded area, 90% confidence interval (CI) of the 5% and 95% critical values; pink-shaded area, 90% CI of 0; red-shaded area, outliers of the bounds of the CI. The M numbers represent the models described in Table 1.

The distribution of the individual CL/F obtained in the post-hoc analysis compared with their theoretical distribution is represented in Figure 1. The distribution of the individual V/F was not performed because half of the models (M3, M4 and M6) did not include IIV in the V/F. The QQ-plot of the NPDE and their distribution versus time are depicted in Figure 2. 

In M2 and M4, the 20% and 80% percentiles of the EBE of CL/F are close to the 95% confidence interval of the 20% and 80% percentiles of the simulated distribution of CL/F for these models. Moreover, the NPDE performed better in these models. Hence, M2 and M4 were the models that best described the studied population, with less bias and better NPDE performance. Therefore, the predictive performance would be evaluated in these models.

### 3.4. Evaluation of Predictive Performance

**Figure 3:**
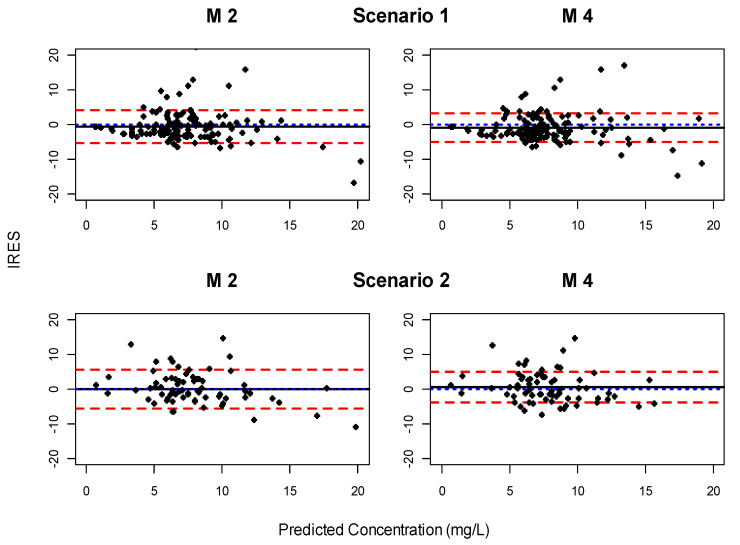
Individual residual (IRES) versus the individual predicted concentrations for M2 and M4 in Scenario 1 and Scenario 2. The mean IRES (black solid line) represents the bias of each model; red dashed line, 5th and 95th percentile for IRES; blue dotted line to highlight the line corresponding to 0. The M numbers represent the models described in Table 1.

Figure 3 shows the predictive performance for M2 and M4 represented as the IRES vs. the model-based prediction of the last observed TSC. Both models behave similarly, with a limited bias and a similar dispersion of the IRES. Table 4 also shows the bias and precision for M2 and M4 and their confidence interval. M2 and M4 are statistically better (*p* < 0.05) than the other models in terms of bias and precision in both scenarios (data not shown). 

### 3.5. Clinical Impact

**Figure 4:**
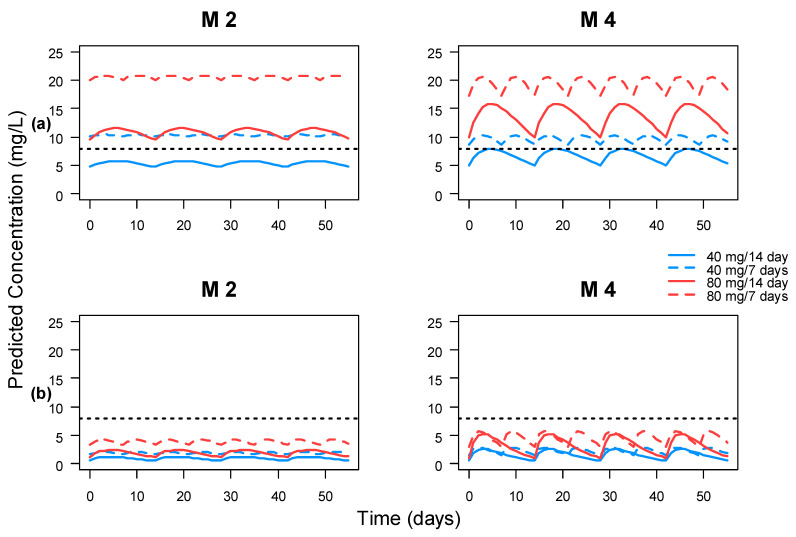
Stochastic simulation of the 25th percentile of serum concentrations over time for M2 and M4. Black dotted line was drawn to highlight the line corresponding to 8 mg/L. (**a**) Serum concentrations of patients without AAA. (**b**) Serum concentrations of patients with AAA. The M numbers represent the models described in Table 1.

The results of the stochastic simulations of different dosage regimens using M2 and M4 are summarized in Figure 4. None of the dosage regimens could reach the desired target (TSC > 8 mg/mL) in at least 75% of the population that developed AAA. Similarly, 40 mg every other week was insufficient to reach the target for at least 75% of the population without AAA, although it is the standard dose recommended by protocol. 40 mg every week or 80 mg every week or every other week are enough to reach the target in at least 75% of the population. Interestingly, according to M2, the plasma concentration profiles of 40 mg every week or 80 mg every other week are very similar, which is not the case in M4.

## 4. Discussion

The MDPI applied in the clinical routine commonly makes use of PopPK models found in literature, given the lack of data available to develop in-house models in most of the hospitals. However, these models must be validated in the target population. An important aspect to validate is the predictive performance of the models, in similar conditions to the clinical routine. Many validations published in the literature do not really validate the predictive performance, but rather evaluate the model adequacy to the data. In the present work, the predictive performance was done with TSC that has not been used to calculate the EBEs, mimicking the real-world scenario. To our knowledge, this is the first validation and comparison of the PopPK models of adalimumab in the literature for their use in clinical routine. Six PopPK models for adalimumab were found in the literature for CD and/or UC patients, with similar structure (one-compartment model), although the covariates included differ among them. The PopPK models included patients with both induction and maintenance treatment, and only one was performed with data from pediatric population.

The model adequacy showed that M2 and M4 performed better than the rest. However, the mean individual CL/F obtained in all six PopPK models after the Bayesian post-hoc estimation (Figure 1) is somehow higher than the expected mean CL/F. One possible explanation for this systematic trend is that the mean albumin value of our population is slightly lower than the referenced in the models found in literature, which indicates a worse disease control. There are several studies that demonstrate the correlation between low levels of albumin and an increase in the clearance of other similar drugs like infliximab [32,33].

Consequently, four out of the six models were discarded due to the significant bias in the distribution of the NPDE as well as the EBEs of the PopPK parameters, therefore, the models M2 and M4 were the candidates to evaluate the predictive performance. The predictive performance of both models performed reasonably well, with a bias less than −0.91, which is less than 13% of the trough target (8 mg/L). The bootstrap analysis of the predictive performance showed no statistical difference between both models, so, with the available data, both models could be considered as equally good for the clinical routine purposes. AAA is considered the covariate with the highest impact in the pharmacokinetic parameters, according to the results of the stochastic simulations.

According to the drug label, the recommended maintenance dose after the induction phase is 40 mg every other week [5]. This scheme results in a mean steady-state TSC of approximately 7 mg/L in Crohn’s disease patients, which agrees with the mean steady-state TSC observed in our population (7.3 mg/L). So far, the exposure target is highly dependent on the therapeutic objective (clinical, endoscopic, biochemical or histologic remission) and whether patients are diagnosed with CD or UC [34]. A recent study showed that patients with concentrations <8.3 mg/L had more risk to develop AAA by week 12 and experienced less clinical benefit from dose escalation due to a loss of response [22]. Another study indicates that 8–12 mg/L TSC of adalimumab are required to achieve mucosal healing in 80–90% of IBD patients [18]. According to the stochastic simulations performed with M2 and M4 and considering a target TSC over 8 mg/mL, the recommended maintenance regimen dosage that should be included in the protocols is 40 mg every week or 80 mg every other week, in order to reach the target in, at least, 75% of the population. These recommendations are in line with the MDPI interventions in our population, where 75% of the patients needed a dose increase to reach the 8 mg/mL target.

The limitation of this study relies on its retrospective design, where patients were selected for MDPI based on the clinical decision of the physician, which implies a bias in the severity of the disease, reflected in the mean albumin values of our population. A prospective study in which patients were included for MDPI in a structured way regardless of the clinical situation of the patients should be carried out to avoid selection bias and validate these results in a wider population.

In summary, two of the PopPK models found in the literature were found to be better than the others in terms of model adequacy and predictive performance. However, the EBEs of the individual CL/F were found biased when compared with the population mean values in the models. That suggested the need to update the model with the available data. On the other hand, the stochastic simulations performed with these models suggested the benefits of increasing the maintenance dose in protocol to reach the 8 mg/L target.

## Figures and Tables

**Table 1:** Summary of specifications of selected models.

Model No.	Study	No. of Patients (Total No. of Samples)	Parameter Values and Covariate Relationships Included	IIV (CV)	Residual Variability
M1	FDA, 2008	646 adult patients (NA)	CL/F (L/h)=0.0127 V/F (L)=9.39+0.126· (WT−72)) ka (1/h)=0.027 FIX	IIV-CL/F: 16.4% IIV-V/F: 35.1%	Prop = 31.6%
M2	Ternant D et al., 2015	65 adult CD patients (341)	CL/F (L/h)=0.0175· (1+4.5 ·AAA) V/F (L)=13.5 ka (1/h)=0.00625	IIV-CL/F: 65% IIV-V/F: 48%	Add = 1.8 mg/L Prop = 16%
M3	Sharma S et al., 2015	189 pediatric CD patients (852)	$\mathrm{CL}/F\;\left(L/h\right)=0.0117\cdot \;\left(1+1.08\cdot \mathrm{AAA}\right)\cdot \;{\left(\mathrm{WT}/45.2\right)}^{0.48}$ $V/F\;\left(L\right)=4.75\;\cdot \;{\left(\mathrm{WT}/45.2\right)}^{0.904}$ ka (1/h)=0.00833	IIV-CL/F: 21.1%	Add = 1.9 mg/L Prop = 7.1%
M4	Berends SE et al., 2018	96 adult CD patients (181)	CL/F (L/h)=0.0133·(1+3.14·AAA)·(1+0.4·DOSING) V/F (L)=4.07 ka (1/h)=0.00833 FIX	IIV-CL/F: 49.1%	Add = 1.02 mg/L Prop = 9%
M5	Vande Castelee et al., 2019	28 adult CD patients (185)	$\mathrm{CL}/F\;\left(L/h\right)=0.01375\cdot \;\left(1+1.59\cdot \;\mathrm{AAA}\right)\cdot \;{\left(\mathrm{LBW}/47.8\right)}^{1.97}$ V/F (L)=7.8 ka (1/h)=0.0143	IIV-CL/F: 32.6% IIV-V/F: 35.6% IIV-ka: 103.9%	Prop = −16.6%
M6	Sánchez-Hernández et al., 2020	104 adult IBD patients (303)	$\mathrm{CL}/F\;\left(L/h\right)=0.0157\cdot \;{\left(\mathrm{BMI}/23.7\right)}^{1.11}\cdot \;\left(1+1.20\cdot \;\mathrm{UDASC}\right)\cdot \;\left(1+0.24\cdot \;\mathrm{PEN}\right)\cdot \;{\left(\mathrm{FCP}/74\right)}^{0.064}$ V/F (L)=11.2 ka (1/h)=0.00625 FIX	IIV-CL/F: 23.2%	Prop = 21.7%

IIV: inter-individual variability; CV: coefficient of variation; CD: Crohn’s disease; IBD: Inflammatory Bowel Disease; WT: weight; AAA: antibodies against adalimumab; DOSING: adalimumab dosing regimen (0: every other week, 1: every week); UDASC: unexplained decline in adalimumab serum concentrations (0: NO, 1: YES); PEN: administration pen device during maintenance phase (0:40 mg, 1:80 mg); FCP: fecal calprotectin; add:additive error; prop: proportional error; NA: not available. The M numbers represent the selected models.

**Table 2:** Summary of patient characteristics of selected models.

Model No.	Age (yr)	Weight (kg)	Disease (cd/uc)	Sex (m/f)	AAA Positive (%)	Albumin (g/dL)	Dosage Regimen	Measured Adalimumab Concentration	Measured AAA
M1	NA	NA	NA	NA	NA	NA	- Induction phase: 160/80 mg or 80/40 at weeks 0/2 - Maintenance phase: 40 every other week	ELISA	ELISA
M2	37 (17–61)	68 (43–109)	100/0	17/48	9 (13.8%)	NA	- Induction phase: 160/80 mg or 80/40 at weeks 0/2 - Maintenance phase: 40 mg every other week	ELISA	Double-antigen ELISA
M3	13.6 (6–17)	45.2 (18–119)	100/0	105/84	83 (43.9%)	4.0 (2.4–5.3)	- Induction phase: ≥40 kg: 160/80 mg at weeks 0/2 <40 kg: 80/40 at weeks 0/2 - Maintenance phase: ≥40 kg: 40 or 20 mg every other week <40 kg: 20 or 10 mg every other week	Double-antigen ELISA	Bridging ELISA
M4	38 (32–44)	65 (58–76)	100/0	35/96	17 (18%)	4.3 (4.05–4.5)	- Maintenance phase: 40 mg every week or every other week	TNF ELISA	Antigen-binding test
M5	37 (30–49)	66 (55–73)	100/0	13/28	5 (17.9%)	3.99 (3.6–4.4)	- Induction phase: 160/80 mg at weeks 0/2 - Maintenance phase: 40 mg every other week	In-house developed TNF-coated ELISA	In-house developed drug resistant AAA assay
M6	43 (32–56)	68 (56–80)	84/20	58/46	0	4.5 (4.3–4.7)	- Induction phase: 160/80 mg at weeks 0/2 - Maintenance phase: dose adjustment according to TDM	ELISA	ELISA

CD: Crohn’s disease; UC: ulcerative colitis; AAA: antibodies against adalimumab; NA = not available. The M numbers represent the models described in Table 1.

**Table 3:** Summary of characteristics of included patients.

Characteristics	Count (%)/Median (Percentile 25th–75th)	Missings, *n* (%)
Patients	134	0
Age (yr)	45 (34–57)	0
Sex, male, *n* (%)	70 (52.2%)	0
Weight (kg)	66 (58–75)	1 (0.75%)
Body mass index (kg/m^2^)	23.85 (20.52–27.36)	10 (7.46%)
Lean Body Weight (kg)	46.84 (42.60–52.10)	10 (7.46%)
Albumin (g/dL)	3.84 (3.53–4.12)	5 (3.73%)
CRP (mg/dL)	0.64 (0.25–2.1)	37 (27.61%)
FCP (mg/kg)	487 (217.11–884.68)	37 (27.61%)
IBD type, CD, *n* (%)	115 (85.8%)	0
Concomitant immunomodulator, *n* (%)		
Aminosalicylate	7 (5.2%)	0
Methotrexate	10 (7.5%)	0
Azathioprine	53 (39.6%)	0
6-Mercaptopurine	6 (4.5%)	0
Corticosteroids	16 (11.9%)	0
Combined	14 (10.4%)	0
Adalimumab serum samples	398	0
Adalimumab serum concentrations (mg/L)	6.75 (4.58–8.65)	0
AAA serum concentrations (mg/L)	29 (4.53–76.30)	0
AAA positive, *n* (%)	11 (8%)	0

CRP: C-reactive protein; FCP: fecal calprotectin; IBD: inflammatory bowel disease; CD: Crohn’s disease; AAA: antibodies against adalimumab.

**Table 4:** Values of bias and precision with its 95% confidence interval for each model in both scenarios.

Model	Scenario 1		Scenario 2	
	Bias	Precision	Bias	Precision
M2	−0.59 (−1.37:0.19)	4.61 (3.55:5.67)	0.012 (−1.27:1.29)	5.43 (3.81:7.06)
M4	−0.91 (−1.62:−0.19)	4.30 (3.47:5.12)	0.52 (−0.52:1.56)	4.43 (3.49:5.37)

The M numbers represent the models described in Table 1.

## Data Availability

The datasets used and/or analyzed during the current study are available from the corresponding author on reasonable request.

## References

[1] Gomollón F., Dignass A., Annese V., Tilg H., Van Assche G., Lindsay J.O., Peyrin-Biroulet L., Cullen G.J., Daperno M., Kucharzik T. (2017). 3rd European Evidence-based Consensus on the diagnosis and management of Crohn’s disease 2016: Part 1: Diagnosis and medical management. J. Crohn’s Colitis.

[2] Baumgart D.C., Sandborn W.J. (2007). Inflammatory bowel disease: Clinical aspects and established and evolving therapies. Lancet.

[3] Bernstein C.N., Eliakim A., Fedail S., Fried M., Gearry R., Goh K., Hamid S., Khan A.G., Khalif I., Ng S.C. (2016). World Gastroenterology Organisation Global Guidelines inflammatory bowel disease. J. Clin. Gastroenterol..

[4] Fakhoury M., Negrulj R., Mooranian A., Al-Salami H. (2014). Inflammatory bowel disease: Clinical aspects and treatments. J. Inflamm. Res..

[5] European Medicines Agency (EMA) (2021). HUMIRA: Summary of Product Characteristics. EMA.

[6] Cohen L.B., Nanau R.M., Delzor F., Neuman M.G. (2014). Biologic therapies in inflammatory bowel disease. Transl. Res..

[7] Roda G., Jharap B., Neeraj N., Colombel J.F. (2016). Loss of response to anti-TNFs: Definition, epidemiology, and management. Clin. Transl. Gastroenterol..

[8] Ma C., Huang V., Fedorak D.K., Kroeker K.I., Dieleman L.A., Halloran B.P., Fedorak R.N. (2014). Adalimumab dose escalation is effective for managing secondary loss of response in Crohn’s disease. Aliment. Pharmacol. Ther..

[9] Yanai H., Lichtenstein L., Assa A., Mazor Y., Weiss B., Levine A., Ron Y., Kopylov U., Bujanover Y., Rosenbach Y. (2015). Levels of drug and antidrug antibodies are associated with outcome of interventions after loss of response to infliximab or adalimumab. Clin. Gastroenterol. Hepatol..

[10] Vande Casteele N., Gils A. (2015). Pharmacokinetics of anti-TNF monoclonal antibodies in inflammatory bowel disease: Adding value to current practice. J. Clin. Pharmacol..

[11] Lopetuso L.R., Gerardi V., Papa V., Scaldaferri F., Rapaccini G.L., Antonio Gasbarrini A., Papa A. (2017). Can we predict the efficacy of anti-TNF-α agents?. Int. J. Mol. Sci..

[12] Ding N.S., Hart A., De Cruz P. (2016). Systematic review: Predicting and optimising response to anti-TNF therapy in Crohn’s disease—algorithm for practical management. Aliment. Pharmacol. Ther..

[13] Papamichael K., Gils A., Rutgeerts P., Levesque B.G., Vermeire S., Sandborn W.J., Vande Casteele N. (2015). Role for therapeutic drug monitoring during induction therapy with TNF antagonists in IBD: Evolution in the definition and management of primary nonresponse. Inflamm. Bowel Dis..

[14] Papamichael K., Vande Casteele N., Ferrante M., Gils A., Cheifetzl A.S. (2017). Therapeutic drug monitoring during induction of antitumor necrosis factor therapy in inflammatory bowel disease: Defining a therapeutic drug window. Inflamm. Bowel Dis..

[15] Papamichael K., Vogelzang E.H., Lambert J., Wolbink G., Cheifetz A.S. (2019). Therapeutic drug monitoring with biologic agents in immune mediated inflammatory diseases. Expert Rev. Clin. Immunol..

[16] Papamichael K., Cheifetz A.S., Melmed G.Y., Irving P.M., Vande Casteele N., Kozuch P.L., Raffals L.E., Baidoo L., Bressler B., Devlin S.M. (2019). Appropriate Therapeutic Drug Monitoring of Biologic Agents for Patients With Inflammatory Bowel Diseases. Clin. Gastroenterol. Hepatol..

[17] Darwich A.S., Ogungbenro K., Vinks A.A., Powell J.R., Reny J.L., Marsousi N., Daali Y., Fairman D., Cook J., Lesko L.J. (2017). Why has model-informed precision dosing not yet become common clinical reality? Lessons from the past and a roadmap for the future. Clin. Pharmacol. Ther..

[18] Ungar B., Levy I., Yavne Y., Yavzori M., Picard O., Fudim E., Loebstein R., Chowers Y., Eliakim R., Kopylov U. (2015). Optimizing anti-TNFα therapy: Serum levels of infliximab and adalimumab associate with mucosal healing in patients with inflammatory bowel diseases. Clin. Gastroenterol. Hepatol..

[19] Papamichael K., Juncadella A., Wong D., Rakowsky S., Sattler L.A., Campbell J.P., Vaughn B.P., Cheifetz A.S. (2019). Proactive therapeutic drug monitoring of adalimumab is associated with better long-term outcomes compared to standard of care in patients with inflammatory bowel disease. J. Crohn’s Colitis.

[20] Brendel K., Comets E., Laffont C., Laveille C., Mentre F. (2006). Metrics for external model evaluation with an application to the population pharmacokinetics of gliclazide. Pharm. Res..

[21] Brendel K., Comets E., Laffont C., Laveille C., Mentre F. (2010). Evaluation of different tests based on observations for external model evaluation of population analyses. J. Pharmacokinet. Pharmacodyn..

[22] Verstockt B., Moors G., Bian S., Van Stappen T., Van Assche G., Vermeire S., Gils A., Ferrante M. (2018). Influence of early adalimumab serum levels on immunogenicity and long-term outcome of anti-TNF naive Crohn’s disease patients: The usefulness of rapid testing. Aliment. Pharmacol. Ther..

[23] Beal S.L., Sheiner L.B., Boeckmann A.J., Bauer R.J. (2021). NONMEM 7.4.0 Users Guides. (1989–2013).

[24] R Core Team (2021). R: A Language and Environment for Statistical Computing.

[25] RStudio (2021). RStudio: Integrated Development Environment for R.

[26] Food and Drug Administration (FDA) (2008). HUMIRA: Clinical Pharmacology and Biopharmaceutics Review(s). *FDA*. https://www.accessdata.fda.gov/drugsatfda_docs/bla/2007/125057_S0089.pdf.

[27] Ternant D., Karmiris K., Vermeire S., Desvignes C., Azzopardi N., Bejan-Angoulvant T., van Assche G., Paintaud G. (2015). Pharmacokinetics of adalimumab in Crohn’s disease. Eur. J. Clin. Pharmacol..

[28] Sharma S., Eckert D., Hyams J.S., Mensing S., Thakkar R.B., Robinson A.M., Rosh J.R., Ruemmele F.M., Awni W.M. (2015). Pharmacokinetics and exposure-efficacy relationship of adalimumab in pediatric patients with moderate to severe Crohn’s disease: Results from a randomized, multicenter, phase-3 study Pharmacokinetics and exposure-efficacy relationship of adalimumab in pediatric patients with moderate to severe Crohn’s disease: Results from a randomized, multicenter, phase-3 study. Inflamm. Bowel Dis..

[29] Berends S.E., Strik A.S., Van Selm J.C., Löwenberg M., Ponsioen C.Y., DʼHaens G.R., Mathôt R.A. (2018). Explaining interpatient variability in adalimumab pharmacokinetics in patients with Crohn’s disease. Ther. Drug Monit..

[30] Vande Casteele N., Baert F., Bian S., Dreesen E., Compernolle G., Van Assche G., Ferrante M., Vermeire S., Gils A. (2019). Subcutaneous absorption contributes to observed interindividual variability in adalimumab serum concentrations in Crohn’s disease: A prospective multicentre study. J. Crohn’s Colitis.

[31] Sánchez-Hernández J.G., Pérez-Blanco J.S., Rebollo N., Muñoz F., Prieto V., Calvo M.V. (2020). Biomarkers of disease activity and other factors as predictors of adalimumab pharmacokinetics in inflammatory bowel disease. Eur. J. Pharm. Sci..

[32] Hemperly A., Vande Casteele N. (2018). Clinical Pharmacokinetics and Pharmacodynamics of Infliximab in the Treatment of Inflammatory Bowel Disease. Clin. Pharmacokinet..

[33] Brandse J.F., Mould D., Smeekes O., Ashruf Y., Kuin S., Strik A., van den Brink G.R., DʼHaens G.R. (2017). A Real-life Population Pharmacokinetic Study Reveals Factors Associated with Clearance and Immunogenicity of Infliximab in Inflammatory Bowel Disease. Inflamm. Bowel Dis..

[34] Juncadella A., Papamichael K., Vaughn B.P., Cheifetz A.S. (2018). Maintenance adalimumab concentrations are associated with biochemical, endoscopic, and histologic remission in inflammatory bowel disease. Dig. Dis. Sci..

